# Risk assessment of daikenchuto-induced hepatobiliary injury in colon cancer patients post-colectomy: a retrospective cohort study

**DOI:** 10.1186/s12906-025-05186-1

**Published:** 2025-12-19

**Authors:** Satoru Watanabe, Yu Wada, Jun Nagata, Takeshi Asakawa, Keiji Hirata, Yoshihisa Fujino

**Affiliations:** 1 Tsumura Advanced Technology Research Laboratories, Tsumura & Co, Ibaraki, 300-1192 Japan; 2 Department of Surgery 1, School of Medicine, University of Occupational and Environmental Health, Japan, Fukuoka, 807-8555 Japan; 3 Department of Environmental Epidemiology, Institute of Industrial Ecological Sciences, University of Occupational and Environmental Health, Japan, Fukuoka, 807-8555 Japan

**Keywords:** Adverse effects, Colectomy, Colon cancer, Herbal medicine, Drug induced liver injury

## Abstract

**Background:**

Colectomy for colon cancer typically results in gastrointestinal hypomotility. Daikenchuto, a herbal medicine traditionally used in Japan, is administered to alleviate gastrointestinal hypomotility. However, it is suspected to cause hepatobiliary injury. Therefore, in this comparative, retrospective cohort study, we aimed to assess the risk of daikenchuto administration-induced hepatobiliary injury post-colectomy.

**Methods:**

Patients with colon cancer who underwent colectomy, excluding the population with a high risk of postoperative hepatobiliary injury, were included in this study (*N* = 17,996). Specifically, patients who received daikenchuto within 4 days after colectomy or from the day of surgery to the first meal postoperatively and those who did not receive daikenchuto were assigned to the daikenchuto exposure and non-exposure groups, respectively. The primary outcome was the postoperative administration of cholagogues or hepatoprotective drugs. Multivariate logistic regression analysis was performed considering the perioperative risk factors for hepatobiliary injury. The analysis estimated the adjusted odds ratios to assess the risk of daikenchuto-induced hepatobiliary injury.

**Results:**

The frequencies of the postoperative administration of cholagogues or hepatoprotective drugs were 52 of 2,324 patients (2.24%) and 421 of 15,672 (2.69%) in the daikenchuto exposure and non-exposure groups, respectively. Furthermore, the adjusted odds ratio and 95% confidence interval (0.62–1.12) were < 1 and included 1, respectively.

**Conclusions:**

No association between daikenchuto and the risk of hepatobiliary injury was identified. Thus, our results imply that daikenchuto can be safely administered post-colectomy.

**Trial registration:**

This study was registered in the Japan Registry of Clinical Trials (Trial ID: jRCT1031240102; https://jrct.mhlw.go.jp/en-latest-detail/jRCT1031240102) on May 22, 2024.

**Supplementary Information:**

The online version contains supplementary material available at 10.1186/s12906-025-05186-1.

## Background

Colectomy is the standard treatment approach for colon cancer. Colorectal cancer is the third most common cancer worldwide [1]. In 2020, colon cancer had the second highest mortality rate among all cancers, and approximately 60% of new colorectal cancer cases were diagnosed as colon cancer [2, 3]. In 2020, Colon cancer accounts for 68% of colorectal cancer cases in Japan [4]. Notably, surgery, chemotherapy, radiotherapy, and targeted therapy are the primary treatment approaches for colon cancer [5]. In Europe, colectomy was performed in 68.4–81.3% of patients with colon cancer between January 1, 2010, and December 31, 2012 [6, 7]. Over 60% of patients with colorectal cancer underwent surgery between 2018 and 2020 in Japan [8].

Postoperative ileus is a major complication of gastrointestinal surgery, with its development associated with the autonomic nervous system and hormones; it is also induced by intestinal manipulation, drugs, and stress [9]. A previous report showed that 13.5% of patients with colon cancer experienced postoperative ileus post-colectomy [10]. Postoperative ileus affects postoperative outcomes and quality of life and inhibits oral intake via inducing gastrointestinal hypomotility [11]. Furthermore, a meta-analysis revealed that medical costs due to postoperative ileus increased to € 7,242 in each patient with colorectal cancer [12].

Notably, daikenchuto (DKT) is widely used in clinical settings to improve gastrointestinal hypomotility. Japanese clinical practice guidelines recommend its administration for early postoperative gastrointestinal hypomotility, and it has been used in clinical settings [13]. DKT, herbal medicine used traditionally in Japan, includes dried compound extracts from three medicinal plants: Japanese Zanthoxylum Peel (*Zanthoxyli piperiti pericarpium*), ginseng (*Ginseng radix*), and processed ginger (*Zingiberis processum rhizoma*) [14]. The effectiveness of DKT in treating gastrointestinal hypomotility has been demonstrated [15–17].

However, DKT is suspected to cause hepatobiliary disorders as a potential adverse effect. Herbal medicine is recognized as the suspected drug that induces liver injury [18]. In a study investigating adverse effects in patients administered herbal medicines, DKT was listed as a suspected agent of drug-induced liver injury [19]. A post-marketing surveillance study of individuals treated with TJ-100 (one of the DKT formulations) reported hepatobiliary injury in 0.3% of the study population [20].

DKT’s effect on the increased risk of hepatobiliary injury post-gastrointestinal surgery remains unclear, and no prior research was available when the current study was conducted. A randomized controlled trial would be ideal for assessing the risk of hepatobiliary injury induced by DKT administration to prevent postoperative ileus. However, conducting such a trial to assess hepatobiliary injury is impossible in a situation where DKT is widely used in clinical settings. Real-world data can aid in answering such research questions. Therefore, in the present study, we aimed to assess the effect of DKT on hepatobiliary injury post-colectomy using large-scale real-world data.

## Methods

### Study design, population exposure, and outcomes

This retrospective cohort study was conducted using patient data provided by Medical Data Vision Inc. (Tokyo, Japan), which compiles administrative claims, electronic medical records, and Diagnosis Procedure Combination (DPC) claims from DPC hospitals (hospitals participating in DPC/Per-Diem Payment System) and commercially provides these data to researchers.

In 2020, the DPC/Per-Diem Payment System was adopted to 1,757 hospitals in Japan [21]. Of the 1,757 DPC hospitals, Medical Data Vision Inc. collected data from 383 DPC hospitals (approximately 27,950,000 patients) in June 2019. Of these, patients with colon cancer (International Classification of Diseases, 10th Edition codes: C186, C187, and C189) who underwent colectomy between April 2008 and May 2019 were selected. Subsequently, patients at high risk of postoperative hepatobiliary injury who met the following criteria were excluded: (i) liver comorbidities; (ii) liver resection on the same day as colectomy; (iii) use of cholagogues and hepatoprotective drugs before the surgery; (iii) complex surgery on the same day as colectomy; (iv) preoperative antibiotics administered; (v) antitumor drugs before the surgery; or (vi) blood transfusion on the day pre-colectomy [22–26]. The follow-up period for each patient was from the colectomy day to the discharge date. For the patients discharged later than 30 days post-surgery, the upper limit of the follow-up period was set to 30 days postoperatively.

In the present study, the evaluated DKT formulation was TJ-100. Specifically, TJ-100 (Tsumura & Co., Tokyo, Japan) accounts for approximately 99% of the inpatient DKT usage between April 2022 and May 2023 [27]. DKT exposure was defined as the first postoperative administration of DKT until day 4 or the day the first meal was served post-colectomy, whichever occurred first. Patients in whom the primary outcome occurred from surgery to DKT administration were assigned to the non-exposure group. However, those exposed to DKT (ethical formulations apart from TJ-100) were excluded.

The primary outcome was the administration of cholagogues or hepatoprotective drugs during the follow-up period in individuals. In contrast, the secondary outcomes were the administration of oral corticosteroids after the occurrence of the primary outcome and the hepatobiliary enzyme abnormality. Hepatobiliary enzyme abnormality was defined as exceeding the criteria of drug-induced liver injury as defined by a consensus report by the Council for International Organizations of Medical Sciences (CIOMS) working group on drug-induced liver injury [28]. The criteria for abnormal serum hepatobiliary enzyme values were defined as follows: alanine transaminase (ALT) >5 times the upper limit of reference value, ALT >3 and total bilirubin (T-Bil) >2 times the upper limit of reference, or alkaline phosphatase (ALP) >2 times the upper limit of reference value. The criteria were suggested to detect severe cases by drug-induced liver injury expert working group, and were superior to others in terms of accuracy, specificity, and positive predictive value [29, 30]. We cited to reference intervals as defined by Japanese Committee for Clinical Laboratory Standards [31]. The upper limits were 1.5 mg/dL, and 322 U/L for T-Bil and ALP, those of ALT were 42 U/L and 23 U/L for male and female, respectively. ALP levels and the reference interval are different from those obtained with current method. As the testing methods for ALP, the IFCC method has been used since 2020, while the JSCC method was used prior to that. The former was an international standard, while the latter was Japan-specific method. Since JSCC method produced higher ALP measurements than the IFCC method, the upper limit of reference interval was also higher than it is at present. Specifically, hepatobiliary enzyme abnormality was assessed only in patients in whom Medical Data Vision Inc. could obtain the result of biochemical testing for hepatobiliary function during the follow-up period. Medical Data Vision Inc. was able to obtain laboratory data from medical institutions that provided consent.

### Patients’ characteristics

We identified the following confounding factors to consider based on previous studies [25, 32–43]: sex, age, body mass index, smoking episode, the total score for activities of daily living on admission, TNM cancer stage, comorbidities, preoperative and postoperative drug use, and blood transfusion within 2 days from the day of surgery. The total score for activities of daily living was defined as the cumulative scores from 10 items divided by 5 based on the Barthel index [44]. This score could range from 0 to 20. TNM cancer stage was defined by the TNM Classification of Malignant Tumors (7–8th Edition) by the Union for International Cancer Control. We identified stroke, heart failure, arrhythmia, angina pectoris, coronary artery disease, ileus, diabetes, and dyslipidemia as the comorbidities. The history of drug use included intestinal motility stimulants, statins, antidepressants, and herbal medicines. Regarding postoperative drug use, data on the days of administration of non-steroidal anti-inflammatory drugs and acetaminophen within 3 days from the surgery were acquired. The patients with missing data for body mass index, smoking episodes, cancer stage, or the total score of activities of daily living were excluded.

### Statistical analysis

A logistic regression model was used to estimate the odds ratio of DKT administration associated with the use of cholagogues or hepatoprotective drugs [45, 46]. Multivariate logistic regression analysis included the individuals’ background (age, sex, body mass index, the total score for activities of daily living, and smoking episode), cancer stage, comorbidities (stroke, heart failure, arrhythmia, angina pectoris, coronary artery disease, ileus, diabetes, and dyslipidemia), preoperative drug use (intestinal motility stimulants, statins, antidepressants, and herbal medicines), blood transfusion, duration of non-steroidal anti-inflammatory drug use, and acetaminophen use, either peroral or intravenous administration. All statistical analyses were performed using R.4.3.0 [47].

The frequencies and upper limit of its Clopper-Pearson 95% confidence interval (CI) of oral corticosteroid administration following the use of cholagogues or hepatoprotective drugs were calculated. Logistic regression was not performed since the frequency in the DKT exposure group was 0.

To explore the effect of DKT on the risk of postoperative hepatobiliary enzyme abnormality, the odds ratio was estimated using the logistic regression model. A multivariate logistic regression included the background, cancer stage, comorbidities, preoperative drug use, duration of non-steroidal anti-inflammatory drug, acetaminophen peroral or intravenous use, and blood transfusion.

## Results

**Fig. 1:**
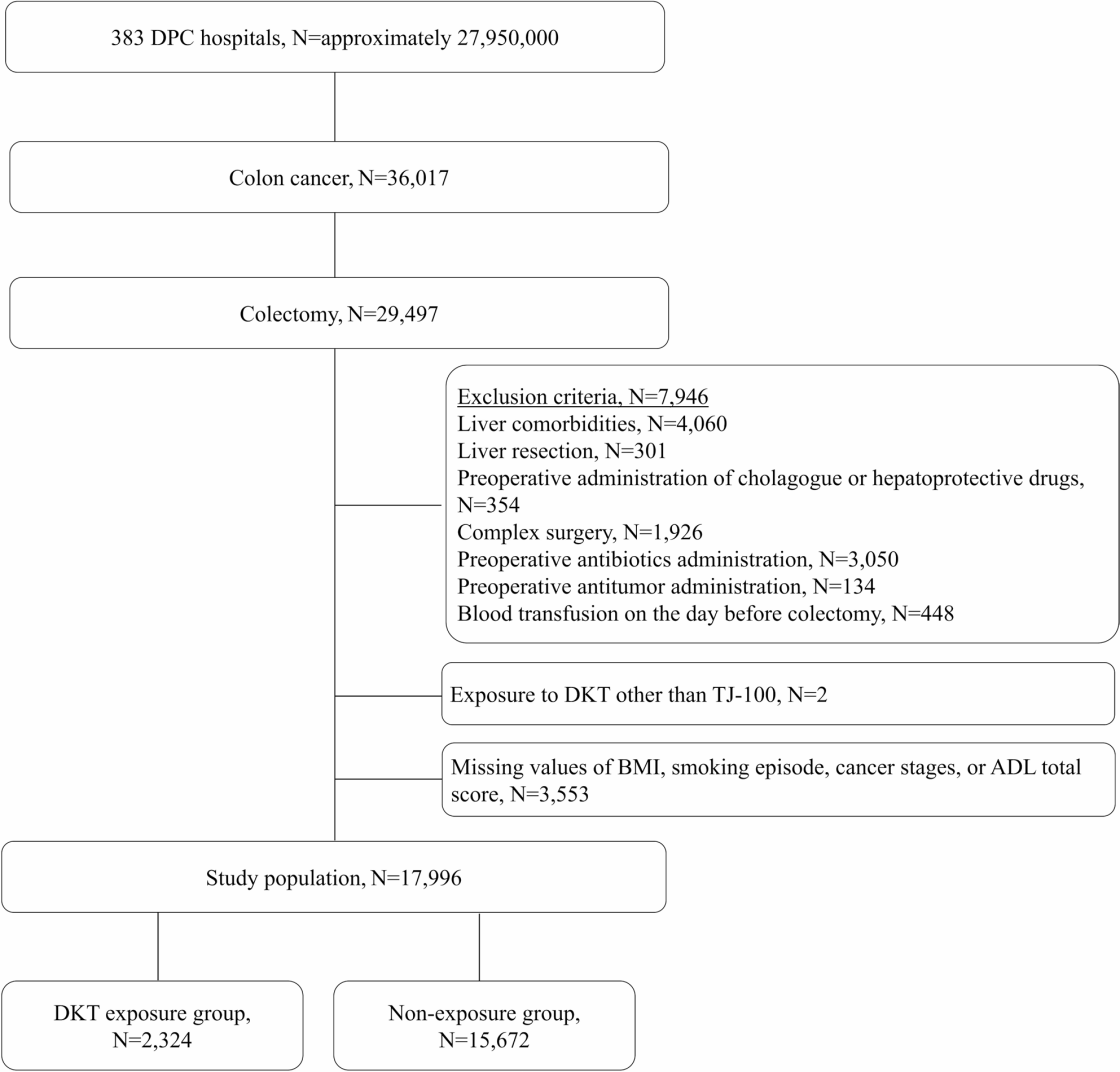
Flowchart of the inclusion and exclusion criteria

Overall, 2,324 and 15,672 patients were assigned to the DKT exposure and non-exposure groups, respectively (Fig. 1). A total of 29,497 of the 36,017 patients with colon cancer underwent colectomy. Furthermore, 7,946 high-risk patients for postoperative hepatobiliary injury were excluded. Two patients exposed to DKT other than TJ-100 and 3,553 with missing data for body mass index, smoking episodes, cancer stages, or total score for the total score for activities of daily living on admission were also excluded. Finally, 17,996 patients were included in this study. Table 1 presents the summary of patients’ backgrounds, cancer stages, comorbidities, drug use, and blood transfusion within 2 days post-surgery.

**Table 1:** Summary statistics of patient characteristics in the two groups

Patient characteristics	DKT exposure group (*N* = 2,324)	Non-exposure group (*N* = 15,672)
Sex ratio (M: F) (% of males)	1,414:910 (60.8)	9,215:6457 (58.8)
Age (years), mean (s.d.)	69.9 (11.2)	69.3 (11.0)
Body mass index (kg/m^2^), mean (s.d.)	23.0 (3.6)	22.9 (3.7)
Smoking episode	1,008 (43.4)	6,459 (41.2)
Total score for activities of daily living, median (range, i.q.r.)	20 (0–20, 20–20)	20 (0–20, 20–20)
Cancer stage		
0	37 (1.6)	377 (2.4)
Ⅰ	756 (32.5)	5,264 (33.6)
Ⅱ	654 (28.1)	4,286 (27.3)
Ⅲ	740 (31.8)	4,702 (30.0)
Ⅳ	137 (5.9)	1,043 (6.7)
Comorbidities		
Stroke	118 (5.1)	793 (5.1)
Heart failure	67 (2.9)	504 (3.2)
Arrhythmia	132 (5.7)	876 (5.6)
Angina pectoris	125 (5.4)	869 (5.5)
Coronary artery disease	204 (8.8)	1,295 (8.3)
Ileus	166 (7.1)	1,135 (7.2)
Diabetes	461 (19.8)	2,997 (19.1)
Dyslipidemia	224 (9.6)	1,615 (10.3)
Drug use and blood transfusion		
Intestinal motility stimulants	142 (6.1)	1,069 (6.8)
Statins	169 (7.3)	772 (4.9)
Antidepressants	28 (1.2)	106 (0.7)
Herbal medicines	196 (8.4)	501 (3.2)
Blood transfusion	150 (6.5)	1,043 (6.7)
Days of non-steroidal anti-inflammatory drug use, median (range, i.q.r.)	1 (0–21, 1–2)	2 (0–33, 1–3)
Days of acetaminophen peroral use, median (range, i.q.r.)	0 (0–23, 0–0)	0 (0–41, 0–0)
Days of acetaminophen intravenous use, median (range, i.q.r.)	0 (0–6, 0–1)	0 (0–7, 0–1)

*DKT* daikenchuto, *M* male, *F* female, *s.d.* standard deviation, *i.q.r.* interquartile range

The values are represented as n (%) unless otherwise indicated

Regarding the primary outcome, 52 of 2,324 patients (2.24%) and 421 of 15,672 (2.69%) in the DKT exposure and non-exposure groups, respectively, were administered cholagogues or hepatoprotective drugs. Multivariate logistic regression analysis showed that the adjusted odds ratio for DKT was 0.84 (95% CI: 0.62–1.12) (Table 2).

**Table 2:** Odds ratios and *P*-values by multivariate logistic regression analyses

	Administration of cholagogues or hepatoprotective drugs		Hepatobiliary enzyme abnormality	
	Odds ratio (CI)	*P*-value	Odds ratio (CI)	*P*-value
Non-exposure group	1.00 (reference)		1.00 (reference)	
DKT exposure group	0.84 (0.62–1.12)	0.260	0.96 (0.52–1.70)	0.907

Each analysis was performed with adjustments for all factors shown in Table 1. DKT, daikenchuto; CI, confidence interval

The frequencies of oral corticosteroid use post-cholagogue or hepatoprotective drug administration were 0 of 2,324 patients (0.00%, upper limit of 95% CI: 0.13%) and 14 of 15,672 (0.09%, upper limit of 95% CI: 0.14%) in the DKT exposure and non-exposure groups, respectively.

Furthermore, the frequencies of hepatobiliary enzyme abnormalities were 15 of 358 patients (4.19%) and 80 of 1,788 (4.47%) in the DKT exposure and non-exposure groups, respectively. The adjusted odds ratio by logistic regression was 0.96 (95% CI: 0.52–1.70). The preoperative enzyme levels for this analysis are summarized in Table S1 in Supplementary materials.

## Discussion

This retrospective cohort study found no relationship between DKT and postoperative hepatobiliary injury in patients with colon cancer who underwent colectomy. To the best of our knowledge, this is the first comparative study to assess DKT’s effect on the risk of postoperative hepatobiliary injury.

We argue that this study provides relatively reliable results. Previous studies have addressed the association between DKT use and liver injury [19, 20]. However, they were observational studies without comparison groups, with few participants, and did not consider confounding factors. Here, we used a large dataset of real-world data with a control group and adjusted for extensive confounding factors. Furthermore, we systematically collected data from 383 acute care hospitals in Japan, which has a relatively small selection bias.

We also assessed hepatobiliary injury based on three outcomes and found no association between the outcomes and DKT administration post-colectomy. First, we defined the administration of the cholagogues and hepatoprotective drugs as the primary outcome. These drugs are administered to improve hepatobiliary function if jaundice or hepatitis are suspected [48, 49]. Second, we defined oral corticosteroid administration as a secondary outcome, which showed no steroid use in the DKT exposure group. Approximately 10% of drug-induced liver injury reportedly progresses to acute liver failure where cases of administration of corticosteroids are considered [50–53]. Third, this study assessed the hepatobiliary injury defined as abnormalities in ALT, T-Bil, and ALP. The CIOMS Working Group on drug-induced liver injury defined the abnormalities in hepatobiliary enzymes as criteria for severe hepatobiliary injury [28]. For example, hepatobiliary enzyme levels exceeding the criteria were observed in patients receiving acetaminophen and non-steroidal anti-inflammatory drugs [38, 39]. Notably, matched results across different outcomes suggest the robustness of this study.

Even from a more conservative perspective, the risk of DKT-induced hepatobiliary injury is assumed to be reasonably minimal. The clinical effect of DKT should be estimated, assuming that the upper limit of the 95% CI for the odds ratio is true. However, under the upper limit of the 95% CI for the odds ratio of 1.12, as shown in Table 2, the frequency of primary outcome in the DKT exposure group is estimated as 3.01% (70 of 2,324 patients). Citing the frequency in the non-exposure group, the risk difference between the two groups is 0.32%. Therefore, the increased risk associated with DKT is, at most, only 0.32%. Minimum detectable odds ratios were computed as a sensitivity analysis. The odds ratios were estimated under the one-upper side z-test by varying the significance rate in the range of 5–99% (the one-sided level of 2.5–49.5%) and powers at 95% or 99%. This analysis shows the detectable odds ratio under the conditions that allow for type 1 error with high power. The minimum odds ratio was estimated as 1.28 (95% power) or 1.42 (99% power) with a significance level of 99% (the one-sided level of 49.5%) (Table 3) [54], suggesting that this study was sufficiently powered to detect a small odds ratio of 1.28. Furthermore, the hepatobiliary risk due to DKT is reasonably small compared with that of other drugs used in clinical settings. Some of the most frequently used drugs in clinical practice pose a high risk of hepatotoxicity. For example, the risks of ibuprofen, piroxicam, and sulindac for liver injury were 1.3, 2.0 and 4.1, respectively [55]. Therefore, these results suggest that the impact of postoperative DKT administration on hepatobiliary function is clinically acceptable in patients undergoing colectomy.

**Table 3:** Detectable odds ratios estimated by sensitivity analyses

Power	Significance level	Minimum detectable odds ratio
0.95	0.05	1.72
	0.10	1.64
	0.20	1.55
	0.30	1.50
	0.40	1.45
	0.60	1.38
	0.80	1.33
	0.99	1.28
0.99	0.05	1.90
	0.10	1.81
	0.20	1.72
	0.30	1.66
	0.40	1.61
	0.60	1.53
	0.80	1.47
	0.99	1.42

This study has some limitations. First, mild hepatobiliary injury that requires no treatment was not assessed. This study assessed moderate and higher hepatobiliary injury of greater clinical importance. Second, the possibilities of confounding that were not included in the data remained. Operative time is related to surgical complications in colectomy [56], and daily intake of alcohol reportedly induces hepatobiliary injury [57]. However, the data provided did not include them. Nonetheless, we excluded typical related factors that may affect the results. Regarding alcohol consumption, alcoholic liver diseases were excluded as liver comorbidities. The medical practice of administrating DKT for cases with long operative times does not exist. Additionally, complex surgery is typical procedure that requires long operative time and has been excluded from study population. In this study, no effect on the result was observed even after accounting for important confounding. Third, the long-term postoperative hepatobiliary outcomes were not assessed, and the occurrence of outpatient complications was unknown. However, postoperative hepatic injury generally occurs within 21 days postoperatively; therefore, the follow-up period in this study is appropriate [58]. Forth, the generalizability of this study to non-Japanese populations may be currently limited. DKT is predominantly used in East Asia; however, in the United States, the development of DKT is underway as TU-100 and the phase 2 clinical trial has been completed (ClinicalTrials.gov ID[NCT04742907]) [59]. When DKT is used overseas in the future, this study will also be beneficial as evidence for safety.

## Conclusions

Our findings revealed that the administration of DKT is unlikely to be a risk factor for postoperative hepatobiliary injury. Thus, our study underscores the safety associated with the perioperative use of DKT and will aid in improving postoperative prognosis in patients undergoing colectomy.

## Supplementary Information

Supplementary Material 1.

## Data Availability

The data that support the findings of this study are available from Medical Data Vision Inc., but restrictions apply to the availability of these data, which were used under license for the current study, and so are not publicly available. Data are however available from the authors upon reasonable request and with permission of Medical Data Vision Inc.

## Abbreviations

ALT: Alanine transaminase
ALP: Alkaline phosphatase
CI: Confidence interval
CIOMS: Council for International Organizations of Medical Sciences
DKT: Daikenchuto
DPC: Diagnosis Procedure Combination
T-Bil: Total bilirubin
