# Supplementary material for: Risk assessment of daikenchuto-induced hepatobiliary injury in colon cancer patients post-colectomy: a retrospective cohort study
Source: BMC Complement Med Ther. 2025 Dec 19;25:442. doi: 10.1186/s12906-025-05186-1 (PMC12717749; doi:10.1186/s12906-025-05186-1)
Supplement: Supplementary file 1 — Supplementary Material 1. [file 12906_2025_5186_MOESM1_ESM.pdf]

Supplementary materials

Risk assessment of daikenchuto-induced hepatobiliary injury in colon cancer patients post-colectomy: A retrospective cohort study

Table S1: Summary of preoperative hepatobiliary enzyme levels in the two groups in the population undergoing assessment for the risk of postoperative hepatobiliary enzyme abnormality.

Preoperative hepatobiliary enzyme levels	DKT exposure group (N = 358)	Non-exposure group (N = 1,788)
alanine transaminase (U/L), geometric mean (GSD)	17.7 (1.7)	17.2 (1.9)
total bilirubin (mg/dL), geometric mean (GSD)	0.6 (1.5)	0.6 (1.6)
alkaline phosphatase (U/L), geometric mean (GSD)	232.0 (1.4)	230.0 (1.4)

46.7% of the individuals were at the preoperative enzyme level. ALP levels and the reference interval are different from those obtained with current method. As the testing methods for ALP, the IFCC method has been used since 2020, while the JSCC method was used prior to that. The former was an international standard, while the latter was Japan-specific method. Since JSCC method produced higher ALP measurements than the IFCC method, the upper limit of reference interval was also higher than it is at present. DKT, daikenchuto; GSD, geometric standard deviation.
